# Screening of feature genes of the ovarian cancer epithelia with DNA microarray

**DOI:** 10.1186/1757-2215-6-39

**Published:** 2013-06-05

**Authors:** Huanchun Ying, Jing Lv, Tianshu Ying, Jun Li, Qing Yang

**Affiliations:** 1 Department of Gynecology and Obstetrics, Shengjing Hospital of China Medical University, No. 36 Sanhao Street, Heping District, Shenyang 110004, China; 2 Department of Oncology, the fifth Hospital of Shenyang, Shenyang 110023, China

**Keywords:** Ovarian cancer, Differentially expressed gene, Cluster analysis, Pathway analysis, Module analysis, Cell cycle

## Abstract

**Objective:**

We aimed to screen differentially expressed genes (DEGs) of ovarian surface epithelia in order to provide beneficial help for early diagnosis and treatment of ovarian cancer with DNA microarrays.

**Methods:**

We extracted the microarray expression profile GSE14407 from Gene Expression Omnibus database which conducted gene expression profiling analysis of 12 ovarian surface epithelia (OSE) and 12 laser capture microdissected serous ovarian cancer epithelia (CEPI) samples. The DEGs between OSE and CEPI were identified by Limma package of R language. Cluster analysis was employed to compare the differences of gene expression patterns between OSE and CEPI. Furthermore, DEGs were analyzed with Functional classification tool, GenMAPP software and GENECODIS.

**Results:**

We identified 1229 DEGs including 592 down-regulated genes and 637 up-regulated genes. Pathway analysis showed that cell cycle was the most significant pathway and the DEGs related with cell cycle were almost up-regulated. Module mining analysis showed that the up-regulated DEGs were related with signal transduction while the down-regulated DEGs were related with lipid metabolism pathway and cytoskeletal structure.

**Conclusion:**

The genes related with cell cycle, lipid metabolism and cytoskeletal structure may be the treatment targets for ovarian cancer.

## Introduction

Ovarian cancer is the fifth most common cause of cancer deaths among women and is the leading cause of death from gynecological neoplastic disease [1]. The average 5-year survival rate is approximately 40%; however, most ovarian cancers are diagnosed when the disease has progressed to the advanced stages III or IV. Patients with advanced disease (stages III and IV) have a significantly lower survival rate of only 10%–20% [2]. A high percentage of mortality results from low diagnosis rate. Survival rates can approach 90% when ovarian cancer is diagnosed at an early stage; however, early detection is challenging, because the relatively nonspecific symptoms of ovarian lesions may be overlooked until abdominal distension by ascites fluid or by large tumor masses becomes unmistakable. Even with extensive surgical debulking and aggressive chemotherapy, the prognosis for women with ovarian cancer currently is not hopeful.

The conventional view is that approximately 90% of ovarian cancers are derived from the single-cell layer of surface epithelium that surrounds the ovary [3]. As the ovarian epithelium transforms into a malignant phenotype, it differentiates into several subtypes that have been categorized into serous, mucinous, endometrioid and clear cell carcinoma, based on their morphology rather than their genotype [4]. Epithelial ovarian cancers show a high degree of genetic heterogeneity as a result of mutations, silencing, and deletions. Since changes in gene expression, either through mutation, epigenetic regulation, or differential splicing events, influence tumor development, progression, drug responsiveness and ultimately the survival of the patient, the identification of the tumor subtype and its genetic fingerprint is essential. Several studies have indicated that different histological subtypes of ovarian carcinoma are associated with different causes and underlying mechanisms, including gene amplification, genetic predisposition, and various carcinogens [5]. Nonetheless, the origin and causes of ovarian carcinoma remain to be elucidated.

The development of microarray technology has provided new insights into cancer diagnosis and treatment. Large-scale microarray studies in breast cancer have succeeded in clarifying 5 molecular subtypes based on gene expression profiles and in developing genomic biomarkers for predicting recurrence in early breast cancer [6]. Thus, breast cancer treatment strategies are being stratified according to molecular characteristics. In contrast, there are no gene expression signatures with high accuracy and reproducibility for clinical diagnosis and management in patients with ovarian cancer because there is a paucity of ovarian cancer samples available for microarray analysis compared with breast cancer. Although TP53 somatic mutation is present in almost all high-grade serous ovarian cancer and plays an important role in the pathogenesis [7,8], high-grade serous ovarian cancer exhibits much biological and molecular heterogeneity that should be considered when developing a novel therapeutic strategy for ovarian cancer [8,9]. A better understanding of the molecular mechanisms leading to ovarian cancer may provide new opportunities for the development of strategies for diagnosis and therapy.

In the present study, we compared the gene expression profile between ovarian surface epithelia (OSE) and laser capture microdissected serous ovarian cancer epithelia (CEPI) samples. Differentially expressed genes (DEGs) were analyzed using gene ontology (GO), molecular pathway, and gene set enrichment analysis algorithms methods. Here we highlight progressive changes that lead to a highly dysregulated cell cycle. These genes, their gene products and the associated signaling pathways may represent novel targets for intervention of ovarian cancer progression.

## Materials and methods

### Source of data

We extracted the microarray expression profile from the study of Nathan J Bowen et al. [10], which was deposited in GEO (Gene Expression Omnibus) database under accession number GSE14407. This study conducted gene expression profiling analysis of 12 OSE and 12 CEPI samples on Affymetrix Human Genome U133 plus 2.0 Array.

### Data preprocessing and DEGs analysis

The probe-level data in CEL files were converted into expression measures and the missing values were imputed [11]. All the data were normalized before statistical analysis [12]. Limma package [13] of R language was used to identify the DEGs between OSE and CEPI. P value was adjusted through the Benjamini and Hochberg procedure [14]. A combination of false discovery rate (FDR) < 0.05 and the absolute value of logFc (Fold change) = 1 was used as the threshold to determine the significance of gene expression difference.

### Comparing the difference of gene expression between OSE and CEPI according to their expression patterns

Cluster analysis for genome-wide expression data from DNA microarray hybridization uses standard statistical algorithms to arrange genes according to similarity in pattern of gene expression. The output is displayed graphically, conveying the clustering and the underlying expression data simultaneously in a form intuitive for biologists. We used cluster analysis for comparing the difference of gene expression patterns between OSE and CEPI samples.

### Gene set enrichment analysis for DEGs

Individual gene analysis (IGA) evaluates the significance of individual genes (approved by Ethical Committee of Shengjing Hospital of China Medical University) between two groups of samples compared. The main problems of IGA originate from the use of the cutoff threshold value. First, the final result of IGA is significantly affected by the selected threshold, which is normally chosen arbitrarily. Second, many genes with moderate but meaningful expression changes are discarded by the strict cutoff value, which leads to a reduction in statistical power. Gene set analysis (GSA) methods free from the problems of the ‘cutoff-based’ methods. GSA directly scores pre-defined gene sets for differential expression and especially aims to identify gene sets with ‘subtle but coordinated’ expression changes that cannot be detected by IGA methods. The key principle is that even weak expression changes in individual genes gathered to a large gene set can show a significant pattern.

Functional Classification Tool used agglomerative clustering algorithm technique for mining the complex function of gene set [15]. We analyzed the up-regulated genes and down-regulated genes separately with cluster method. FDR < 0.05 was set as threshold.

### Pathway analysis for DEGs

GenMAPP [16] (Gene Map Annotator and Pathway Profiler) is a free, open-source, stand-alone application for visualizing, analyzing and sharing genome-scale data in the context of biological pathways. GenMAPP allows users to view and analyze genome-scale data, such as microarray data, on biological pathways, GO terms or any other grouping of genes. In the present study, GenMAPP was used to identify which pathways are affected by analyzing DEGs in ovarian cancers.

### Module mining and function analysis for DEGs

Gene sets reflect biological modules only approximately. Only a subset of genes in a set may contribute to its expression signature, and different gene sets may have similar signatures across the arrays, owing to either an overlap between the gene sets or co-regulation of non-overlapping gene sets. When several gene sets (a cluster) have similar signatures, we extracted from this cluster a core module, which both refines the gene composition of each gene set and combines several related gene sets. This module more closely reflects the genes that participate in a specific biological process, as it consists of the genes whose expression profile corresponds to the signature of the cluster [17].

We used GeneCodis software to integrate DEGs to find groups of genes with similar biological meaning. GeneCodis [18], a web-based application, is a tool for singular and modular enrichment analysis that integrates information of diverse nature (e.g. functional, regulatory or structural) by looking for frequent patterns in the space of annotations and computing their statistical relevance. It can provide analysis of different annotations, including the three GO categories (biological process, cellular component, and molecular function), KEGG pathways, InterPro Motifs, and Swiss-Prot keywords. This integrative capacity sheds light on different aspects of the same information and provides a more accurate interpretation of the data.

## Results

### DEGs analysis

We obtained publicly available microarray dataset GSE14407 from GEO database. After preprocessing and normalization, at the threshold of FDR < 0.05 and |logFC| > 1, we got 1229 DEGs which included 592 down-regulated genes and 1637 up-regulated genes.

### Differential gene expression patterns between OSE cells and CEPI

**Figure 1:**
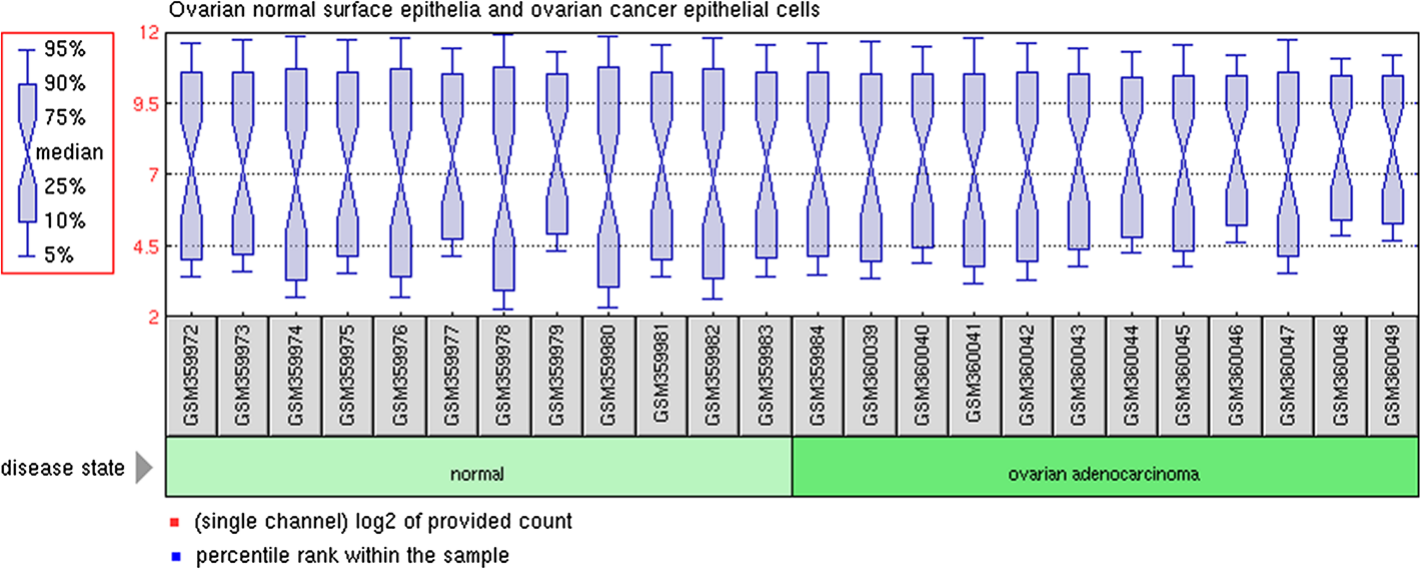
**The box plots generated using the normalized data of 12 ovarian surface epithelia (OSE) and 12 ovarian cancer epithelia (CEPI) samples.** OSE and CEPI samples are indicated with light green and deep green boxes respectively. Black line in each box represents the median of each sample. All the black lines are almost in the same position indicating minimum variability in these datasets.

The hierarchical clustering algorithm was used to compare the differential gene expression patterns between OSE and CEPI samples. The clustered images in Figure 1 is the presence of large contiguous patches of color representing groups of genes that share similar expression patterns. From the colored Figure, we could see that there were significant gene expression differences between OSE and CEPI samples.

### Gene set enrichment analysis for DEGs

**Figure 2:**
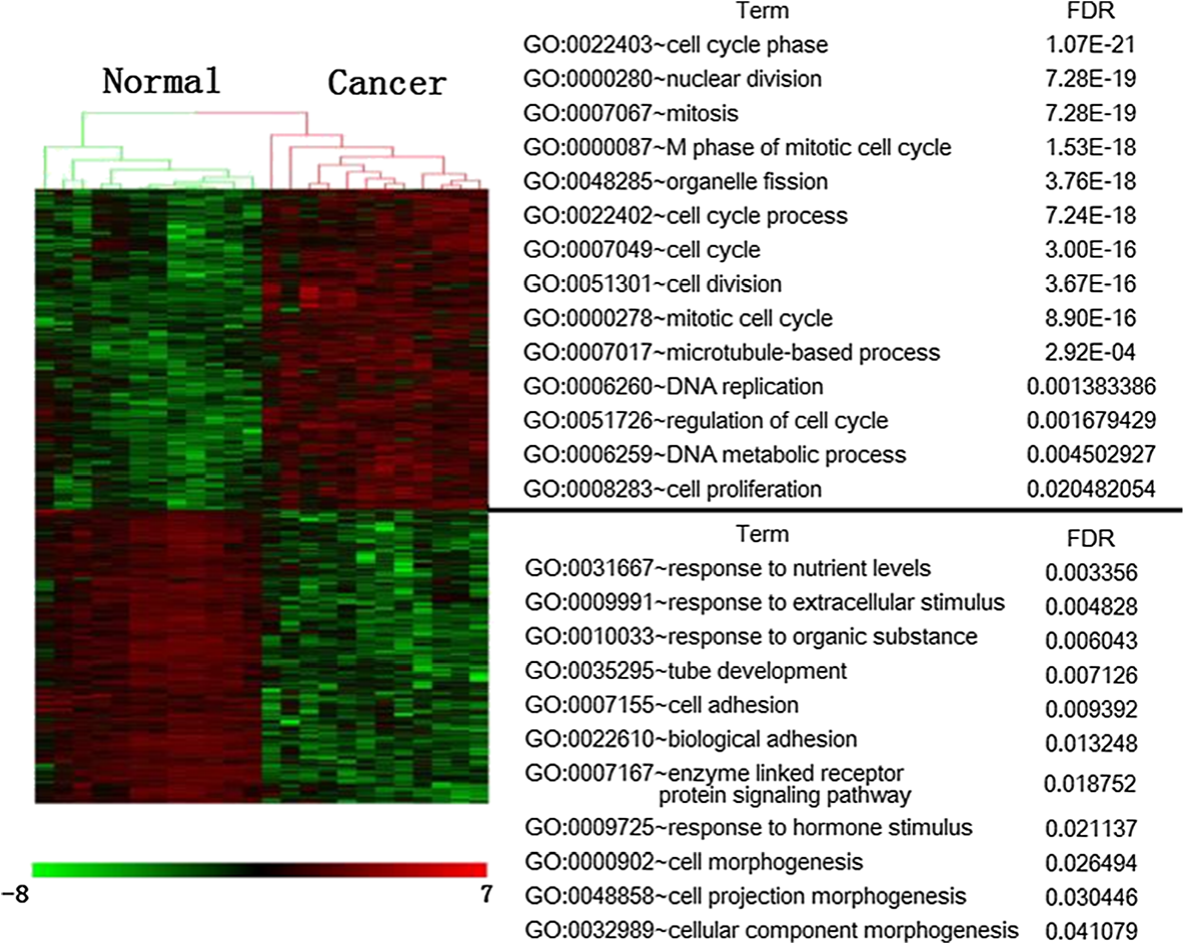
**Hierarchical clustering and gene ontology (GO) enrichment of DEGs between ovarian surface epithelia (OSE) and ovarian cancer epithelia (CEPI) samples.** The heat map (left) was generated by Z-score normalization of log2 expression values from Affymetrix HGU133 Plus 2.0 3 to display the relative expression levels of genes (rows) differentially expressed (red = relatively upregulated; green = relatively downregulated) in 12 OSE and 12 CEPI samples (columns). Uniquely, enriched GO terms are listed for each set of DEGs and their statistical significances corrected by false discovery rate (FDR), hypergeometric distribution p-values.

We mapped all the DEGs into Functional Classification Tool and performed functional enrichment analysis. The results showed that most of the up-regulated DEGs in CEPI were related with cell cycle (Figure 2, right side).

### Pathway analysis for DEGs

**Figure 3:**
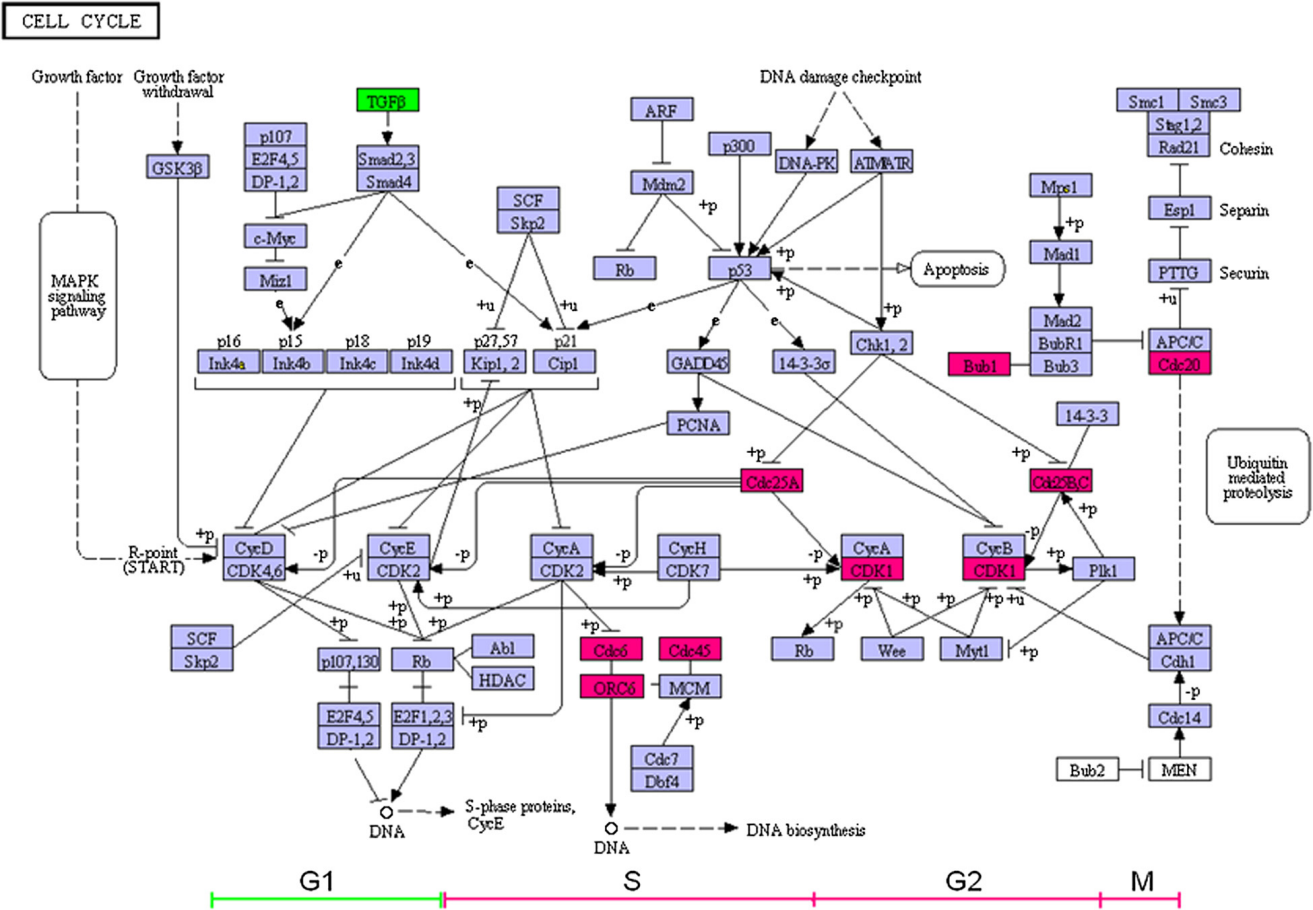
**A GenMAPP schematic of cell cycle pathway genes.** Genes significantly overexpressed in ovarian cancer epithelia samples (CEPI) relative to ovarian surface epithelia (OSE) samples are colored red. The execution of the cell cycle is depicted from left to right. Genes involved in maintaining G1 are generally donregulated (Green) while genes involved in G1 to S progression, G2, and M are upregulated (Red) in CEPI.

GenMAPP pathways, called MAPPs, contain a set of gene or protein identifiers as well as graphical elements, with the custom layouts depicting the relationships between genes and proteins. We mapped all the DEGs into GenMAPP and obtained the most significant pathway- cell cycle which included 9 up-regulated DEGs and 1 down-regulated DEGs (Figure 3). The only one down-regulated DEG was in G1 phase. The DEGs in S, M, G2 phase were all up-regulated.

### Module mining and function analysis

**Figure 4:**
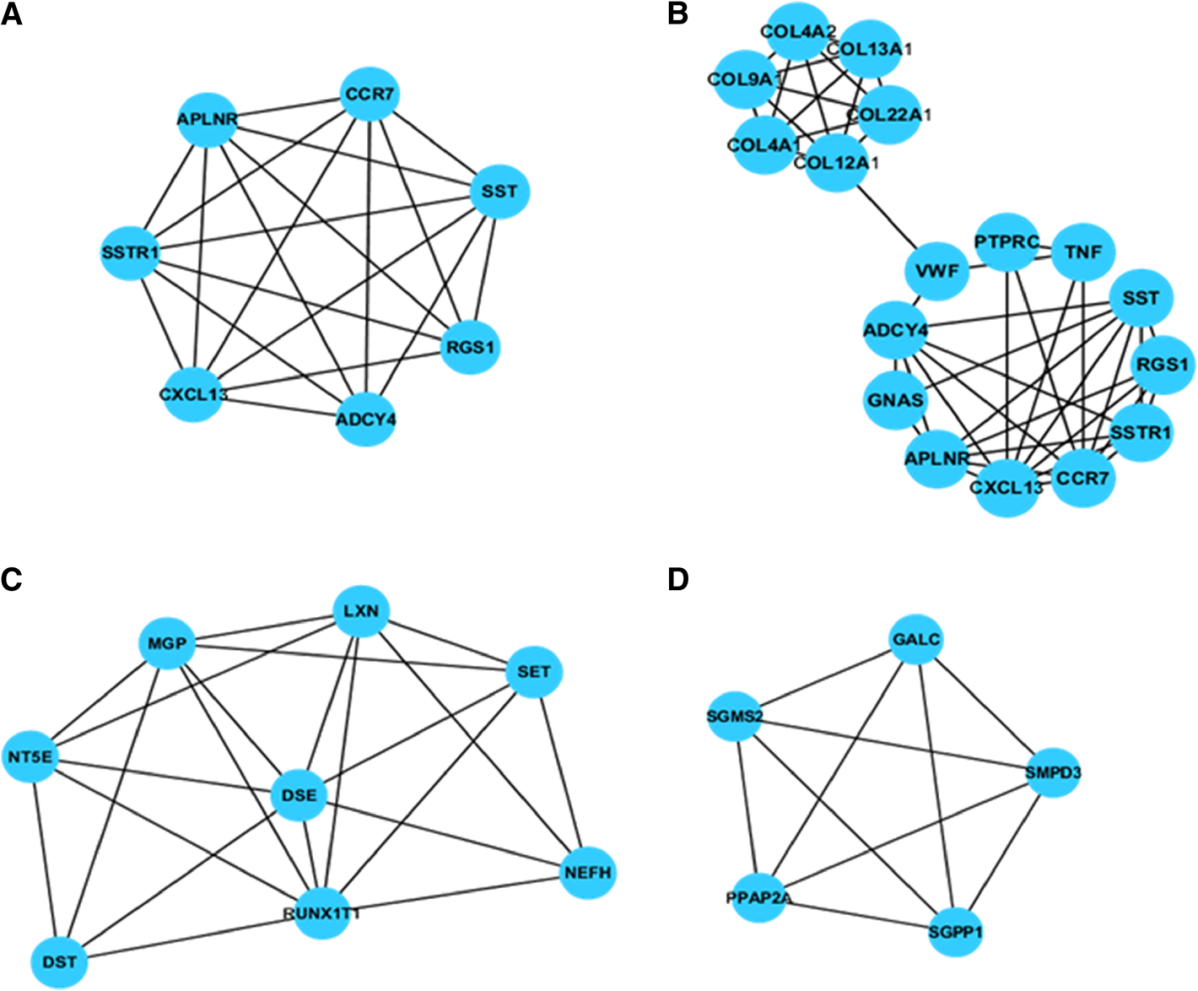
**Module mining results. A** and **B** are modules containing up-regulated DEGs; **C** and **D** are modules containing down-regulated DEGs.

The down-regulated DEGs and up-regulated DEGs were mapped into GENECODIS separately and four modules were obtained individually (Figure 4). The up-regulated DEGs in the 2 modules (Figure 4A and 4B) were related with signal transduction), while the down-regulated DEGs in the other 2 modules (Figure 4C and 4D) were related with lipid metabolic process and cytoskeletal structure (Table 1).

**Table 1:** **Functional annotations for genes in modules A**, **B**, **C and D**

**Module**	**GO-ID**	**Corr p-value**	**N**	**Description**
A	7187	1.33E-03	3	G-protein signaling, coupled to cyclic nucleotide second messenger
	19932	3.69E-03	3	Second-messenger-mediated signaling
	9605	2.83E-03	4	Response to external stimulus
	7186	1.64E-04	5	G-protein coupled receptor protein signaling pathway
	7166	3.34E-03	5	Cell surface receptor linked signaling pathway
	42221	4.01E-03	5	Response to chemical stimulus
	23033	9.18E-03	5	Signaling pathway
	23052	8.46E-03	6	Signaling
	50896	9.18E-03	6	Response to stimulus
	19932	1.94E-03	5	Second-messenger-mediated signaling
B	7187	1.94E-03	4	G-protein signaling, coupled to cyclic nucleotide second messenger
	7186	1.94E-03	6	G-protein coupled receptor protein signaling pathway
	19935	1.94E-03	4	Cyclic-nucleotide-mediated signaling
	7166	3.96E-03	8	Cell surface receptor linked signaling pathway
	1958	9.43E-03	2	Endochondral ossification
C	45104	0.0069735	2	Intermediate filament cytoskeleton organization
	45103	0.0069735	2	Intermediate filament-based process
D	6665	3.9682E-07	4	Sphingolipid metabolic process
	6643	3.9682E-07	4	Membrane lipid metabolic process
	44255	3.6038E-06	5	Cellular lipid metabolic process
	46519	0.000014759	3	Sphingoid metabolic process
	6629	0.000017443	5	Lipid metabolic process
	6644	0.00036681	3	Phospholipid metabolic process
	19637	0.00038844	3	Organophosphate metabolic process

N: number of enriched genes; Corr p-value: p value was adjusted by Benjamini and Hochberg procedure.

## Discussion

In the present study, we have identified genes and their functional categories that are altered in CEPI samples. The gene expression profiles displays statistically significant changes in cell cycle, signal pathway, metabolism and other functional categories that correspond well with many of the morphological changes and biological behaviors.

Dysregulation of the cell cycle is a hallmark of many cancers and control and timing of the cell cycle involves checkpoints and regulatory pathways that ensure the fidelity of DNA replication and chromosome segregation [19]. These processes involve a large collection of key molecules, which are excellent candidates for ovarian cancer susceptibility variants. These include the cyclins (CCNA1, CCNA2, CCNB1, CCNB2, CCND1, CCND2, CCND3, CCNE1, CCNE2, CCNG1, CCNG2), cyclin-dependent kinases (CDKS: CDK1, CDK2, CDK4, CDK6, CDK7, CDC2), CDK inhibitors (CDKN1A, CDKN1B, CDKN2A, CDKN2B, CDKN2C, CDKN2D) and CDC2 regulators (CDC25A, CDC25B, CDC25C) [19]. In the current study, CDK1, CDC6, CDC20, CDC25A, CDC25B, CDC25C, CDC45, Bub1, ORC6 were up-regulated. Studies have shown that up-regulation of CDC6, CDC 20, CDC25 family and Bub1 has relationship with human cancers.

Bub1 encodes a kinase involved in spindle checkpoint function. Several studies have found high Bub1 levels in subsets of breast and gastric cancers, and lymphomas. Furthermore, independent studies of diverse tumor types have identified Bub1 as a gene whose up-regulation correlates with poor clinical prognosis [20].

It is reported that Cdc20 functions as an oncoprotein to promote the development and progression of human cancers [21]. Microarray studies have recently reported overexpression of CDC20 in various tumors, such as tumors of the oral cavity [22], stomach [23], brain (glioblastoma) [24], urinary bladder [25], uterine cervix [26], and ovary [27]. Meta-analysis of cancer microarray data also identifies CDC20 as one of the highly expressed genes in various human cancer tissues [28]. Furthermore, expressions of specific genes including CDC20 consistently correlate with total functional aneuploidy and are predictive of poor prognosis in several cancer types using a computational method [29]. Particularly in serous epithelial ovarian cancer, overexpression of CDC20 is a prognostic factor associated with clinical stage of disease, irrespective of tumor grade [27].

The cell division cycle 25 (CDC25) families of proteins is a group of highly conserved dual-specificity phosphatases. There are three isoforms: CDC25A, CDC25B and CDC25C. They are key regulators of normal cell division and the cell response to DNA damage, and play a fundamental role in transitions between cell cycle phases during normal cell division, via the activation of CdK/cyclin complexes. Their abnormal expression, detected in a number of tumors, often correlated with a poor clinical prognosis, implies that their dysregulation is involved in malignant transformation [30]. In the context of the progression of cell division, the A and B isoforms of CDC25 have been reported as potential oncogenes, being overexpressed in more than ten types of human cancer, including prostate and breast cancers, as well as vulvar squamous cell carcinomas. In contrast, CDC25C is expressed at a far lower level in a limited number of tumors [30].

Module analysis showed that the modules containing the down-regulated DEGs were related with lipid metabolic process and cytoskeletal structure. The modules containing up-regulated DEGs were related with signal transduction. In the last decade, the altered lipid metabolism has increasingly been recognized as another common property of malignant cells [31,32]. Like glucose metabolism, lipid metabolism in cancer cells is also regulated by the common oncogenic signaling pathways, and is believed to be important for the initiation and progression of tumors [32]. Some newly generated lipids molecules, such as phosphatidic acid (PA), diacylglycerol (DAG), and lysophosphatidic acid (LPA), also mediate signal transduction in cancer cells [32]. These lipids regulate a variety of cellular functions including cell proliferation, survival and migration by either activating other signaling proteins inside the cells, or binding to a series of G protein-coupled receptors (GPCRs) on the cell surfaces. It has been reported that inhibitors of lipid metabolic pathways, particularly drugs targeting the mevalonate pathway, have been suggested to be valuable in enhancing the effectiveness of epidermal growth factor receptor-tyrosine kinase inhibitors (EGFR-TKIs) [33]. On the other hand, oncogenic signaling pathways can regulate lipid metabolism at multiple steps, including transcriptional, translational and post-translational levels.

The cytoskeleton plays an important role in tumor cell progression and events such as migration and invasion, allowing the cells to adapt and survive in different microenvironments; compounds that regulate cytoskeleton organization have been used as cancer therapeutics [34]. On the other hand, the organization of the cytoskeleton affects cellular organization, adhesion complexes and polarity, and vesicular transports. Creekmore et al. demonstrate that cytoskeleton disorganization can have profound effects on the subcellular localization of important signaling intermediates, which ultimately may lead to modulated signaling pathways contributing to ovarian cancer development [4].

The complex molecular processes underlying the onset and development of epithelial ovarian cancer is only beginning to be unraveled. Our results indicate that cell cycles, lipid metabolic pathways, cytoskeleton changes and some signal transduction pathways are involved in the establishment and development of ovarian cancer. While many of these pathways have previously been either directly or indirectly implicated in ovarian cancer, detailed network analyses of our gene expression data led to the identification of linkages between these pathways attributable to the altered expression of key regulatory genes.

### Synopsis

Our results indicate cell cycles, lipid metabolic pathways, cytoskeleton changes and some signal transduction pathways are involved in the establishment and development of ovarian cancer.

## Competing interest

All authors have no conflict of interest to declare.

## Authors’ contributions

HCY and JLV articipated in the design, analyses and data interpretation and drafted the manuscript. JL and QY helped to retrieve pathologic and clinical information and provide valuable insight during manuscript preparation. All authors read and approved the final manuscript.
